# Changes in brain functional networks in remitted major depressive disorder: a six-month follow-up study

**DOI:** 10.1186/s12888-023-05082-3

**Published:** 2023-08-28

**Authors:** Jiaqi Zhong, Jingren Xu, Zhenzhen Wang, Hao Yang, Jiawei Li, Haoran Yu, Wenyan Huang, Cheng Wan, Hui Ma, Ning Zhang

**Affiliations:** 1 Affiliated Nanjing Brain Hospital of Nanjing Medical University, No.264 Guangzhou Street, Gulou District, Nanjing, 210029 Jiangsu China; 2 Department of Medical Informatic, School of Biomedical Engineering and Informatics, Nanjing Medical University, Nanjing, 210029 Jiangsu China; 3 Functional Brain Imaging Institute of Nanjing Medical University, Nanjing, 210029 Jiangsu China; 4 Cognitive Behavioral Therapy Institute of Nanjing Medical University, Nanjing, 210029 Jiangsu China; 5 School of psychological and cognitive sciences, Peking University, Beijing, 100871 China

**Keywords:** Central executive network, Salience network, Default mode network, Remitted major depressive disorder, Psychosocial functioning

## Abstract

**Background:**

Patients with remitted major depressive disorder (rMDD) show abnormal functional connectivity of the central executive network (CEN), salience networks (SN) and default mode network (DMN). It is unclear how these change during remission, or whether changes are related to function.

**Methods:**

Three spatial networks in 17 patients with rMDD were compared between baseline and the six-month follow-up, and to 22 healthy controls. Correlations between these changes and psychosocial functioning were also assessed.

**Results:**

In the CEN, patients at baseline had abnormal functional connectivity in the right anterior cingulate, right dorsolateral prefrontal cortex (DLPFC) and inferior parietal lobule (IPL) compare with HCs. There were functional connection differences in the right DLPFC and left IPL at baseline during follow-up. Abnormal connectivity in the right DLPFC and medial prefrontal cortex (mPFC) were found at follow-up. In the SN, patients at baseline had abnormal functional connectivity in the insula, left anterior cingulate, left IPL, and right precuneus; compared with baseline, patients had higher connectivity in the right DLPFC at follow-up. In the DMN, patients at baseline had abnormal functional connectivity in the right mPFC. Resting-state functional connectivity of the IPL and DLPFC in the CEN correlated with psychosocial functioning.

**Conclusions:**

At six-month follow-up, the CEN still showed abnormal functional connectivity in those with rMDD, while anomalies in the SN and DMN has disappeared. Resting-state functional connectivity of the CEN during early rMDD is associated with psychosocial function.

**Clinical trials Registration:**

Pharmacotherapy and Psychotherapy for MDD after Remission on Psychology and Neuroimaging. https://www.clinicaltrials.gov/, registration number: NCT01831440 (15/4/2013).

## Background

Major depressive disorder (MDD) is a mental disorder characterized by significant, persistent depressed mood and cognitive changes [1]. MDD creates significant mental health burdens for the patient and society, and is a main cause of mental health disability [2]. MDD is also the most common global mental disorder, affecting nearly 350 million people [3]. Remitted MDD (rMDD) describes patients who met MDD criteria (e.g., < 7 on the 17-item Hamilton Rating Scale for Depression [HAMD]) [4] and who, after treatment (e.g., antidepressant, behavioral therapy), have improved depressive symptoms to the extent that they no longer meet the MDD diagnostic criteria [5]. While remission is of great significance in antidepressant treatment, it is largely controversial. Symptom-based diagnostic criteria may not accurately represent relief from MDD [6–8], patients with MDD still face high recurrence rates [9] and psychosocial dysfunction and abnormal brain functions pay persist [8, 10, 11].

MDD has been associated with structural and functional brain abnormalities [12, 13]. Resting-state brain functional network connectivity can effectively predict which symptoms will improve, and their degree of improvement, with antidepressant treatment [14, 15]. FC changes in brain functional networks are also important indicators of MDD dysfunction. Pre-remission studies have revealed abnormalities in three specific networks. The central executive network (CEN), salience network (SN), and default mode network (DMN) are core networks associated with MDD-based clinical characteristics, including emotion and cognition [16].

The CEN includes the dorsolateral prefrontal cortex (DLPFC), inferior parietal lobule (IPL), dorsal anterior cingulate cortex, and other regions. In most studies, patients with MDD have reduced CEN activity [17–19] and this region changes during the MDD treatment process [20, 21].

The SN includes the insula, amygdala, anterior cingulate gyrus, DLPFC, medial prefrontal cortex (mPFC), ventrolateral prefrontal cortex, and precuneus [22–24]. Low FC of the insula within the SN network in MDD is associated with forced self-attention [25], a significant clinical feature of MDD [26]. High active SN levels are associated with attention to negative stimuli [27], allowing patients with MDD to focus on threat stimuli more easily. The SN also plays an important role in predicting MDD treatment effects [28].

The DMN includes the mPFC, posterior cingulate gyrus, hippocampus, and temporal lobe [29]. Compared with healthy controls, patients with MDD have increased FC in the anterior medial cortex and decreased FC in the posterior medial regions of the resting-state DMN [30].

Among patients with rMDD, the CEN and SN are overactivate when recognizing emotion-related images, possibly indicating residual dysfunction [31, 32]. While abnormal DMN brain FC in patients with MDD is reduced post-remission, it does not recover to the levels of healthy controls [33]. Although few studies have been conducted with patients with rMDD, these data support the hypothesis that abnormal network functions change rather than disappear. They also support the use of brain function networks for exploring the MDD remission process.

While, as noted above, symptom remittance may not accurately reflect general improvement among patients with MDD, it may be better reflected by functional recovery [8]. However, current MDD-related studies have focused on depressive symptoms and paid less attention to psychosocial functional impairment [34–36]. In post-treatment remission, although rMDD symptoms may be subthreshold, functional abnormalities such as cognitive dysfunction, physical symptoms, and emotional distress may persist [35, 37–39]. These abnormalities affect patients’ lives, causing unemployment and interpersonal relationship problems [10, 35, 40]. Follow-up studies have shown residual psychosocial dysfunction six months post-remission, with gradual recovery [41–43]. Psychosocial recovery among patients with rMDD may also plateau after six months [44]. Neural network abnormality may be one explanation for social functional abnormalities in rMDD [45]. Brain functional networks have been associated with psychological functions, especially cognitive functions such as working memory, cognitive control, self-processing, and social cognition [46–49]. Evaluating whether these networks are associated with psychosocial dysfunction in rMDD is novel, and the results of this approach have potential clinical implications.

In sum, brain functional network abnormalities may persist in patients with rMDD, with negative impacts. As few studies have evaluated functional networks in rMDD, it is unclear how they change during remission and whether such abnormalities are related to psychosocial dysfunction. Thus, the study aim was to explore this potential recovery mechanism and assessment method in rMDD, to track changes in these processes over time and to determine the relations between ongoing brain functional network abnormalities and psychosocial functioning. According to previous studies, it can be assumed that abnormalities in brain functional networks among those with rMDD, which may be related to psychosocial function, may persist and gradually disappear during remission.

Herein, resting-state functional magnetic resonance imaging (rs-fMRI) was used to explore abnormal FC in patients with rMDD. This task-free neuroimaging method eliminates some of the confusion associated with performance, providing a reliable way to measure baseline brain activity and resting-state FC (rs-FC) [50]. Specifically, the CEN, SN, and DMN were analyzed using independent component analysis (ICA).

## Materials and methods

### Participants

Forty-seven participants were included in the rMDD group, among whom 19 were lost to follow-up from address change, lost contact, or personal reasons. Seven of the original participants relapsed during follow-up and 21 completed the six-month follow-up. Among these, 4 were later removed due to head movements during MRI, leaving a final 17 participants for inclusion in analyses (7 male, 10 female). Twenty-two healthy controls (HCs) were also included (14 males, 8 females).

This study was approved by the medical ethics committee of the Brain Hospital affiliated with Nanjing Medical University (ethical approval number: 2015-ky002). All participants participated voluntarily and had signed written informed consent.

#### rMDD group

All patients were hospitalized in the Department of Medical Psychology, Nanjing Brain Hospital, which is affiliated with Nanjing Medical University, between October 2015 and May 2018. Participants were enrolled in the study within a week of meeting its criteria. Patients who met termination criteria were screened out during follow-up. All participants were treated pharmaceutically and such treatments were maintained during remission. All assessments and diagnoses were verified by two psychiatrists.

The inclusion criteria included the following: (1) met standard MDD clinical criteria in the Diagnostic and Statistical Manual of Mental Disorders (DSM)-IV-TR (Structured Clinical Interview for DSM-IV Axis I Disorders, Patient Edition) and had reached the standard for clinical cure (score < 7 on the 17-item HAMD for at least two weeks) after antidepressant treatment; (2) were aged 18–55 years; (3) were right-hand dominant; and (4) were voluntary participants who had signed informed consent.

The exclusion criteria were: (1) comorbid severe physical or infectious disease; (2) other psychiatric disorder; (3) electroconvulsive therapy or modified electroconvulsive therapy during hospitalization and/or systematic psychotherapy during treatment; (4) drug and/or alcohol dependence; and (5) contraindication for MRI.

The termination criteria were: (1) voluntarily stopping medication(s); (2) relapse or manic episode; (3) reluctance to continue; and (4) experiencing a serious life change during follow-up.

#### Healthy control group

Healthy participants who met the healthy group version of the MDD standard clinical criteria in the DSM-IV-TR (Structured Clinical Interview for DSM-IV Axis I Disorders, Non-Patient Edition) were publicly recruited from October 2015 to May 2018. All assessments and diagnoses were verified by two psychiatrists. The inclusion criteria were: (1) aged 18–55 years; (2) right-hand dominant; (3) volunteer and had signed the informed consent. The exclusion criteria were: (1) serious physical disease; (2) personal and/or family history of mental illness; and (3) contraindication for MRI.

### Demographics and self-assessments

All participants completed the general information form, which included age, years of education, and gender.

Psychosocial functioning of patients with rMDD was assessed using the psychological and social functioning subscales of the comprehensive Generic Quality of Life Inventory (GQOLI-74) questionnaire, which can be used to assess quality of life among unique populations (e.g., older adults, patients who are chronically ill). The instrument evaluates 20 factors across four dimensions, including psychological and social functioning. The questionnaire has high reliability and validity, and its test-retest reliability is 0.84–0.93.

Self-rating scales have higher sensitivity and discrimination for symptom assessment [7]. Thus, self-rated symptoms were assessed among the patients with rMDD with the Beck Depression Inventory (BDI), which is commonly used to measure depressive symptoms in MDD, including depressed mood, negative thinking, and suicidal thoughts.

The rMDD group completed the GQOLI-74 subscales and BDI at baseline and six-month follow-up. All assessments were attended by a researcher to ensure that the participant understood their instructions and completed them carefully.

### rs-fMRI date acquisition

All images were obtained on a Siemens 3.0-Tesla signal scanner (Siemens, Verio, Germany) with a standard head coil, at Nanjing Brain Hospital. During the scanning process, foam pads and earplugs were used to reduce head movement and noise, respectively. Participants were instructed to close their eyes, stay awake, and not engage in any mental exercises. A gradient-recalled echo-planar imaging pulse sequence was used for rs-fMRI date acquisition, with the following settings: repetition time (TR)/echo time (TE) = 3000 ms/40 ms, flip angle = 90°, slice thickness = 4.00 mm, field of view = 240 mm×240 mm, and matrix size = 64 × 64. The scan lasted 5.06 min. A high-resolution T1-weighted magnetization prepared gradient echo image was obtained for each participant, for spatial normalization and localization, with the following parameters: TR = 1900 ms, TE = 2.48 ms, slice thickness = 1.00 mm, gap = 0.5 mm, slice number = 176, and matrix size = 256 × 256. The resting-state scan lasted 4.3 min. Rs-fMRI data were collected from the rMDD and HC groups at baseline. Six-month follow-up rs-fMRI data were collected for only the rMDD group.

### Statistical analysis

Rs-fMRI data were preprocessed with Statistical Parametric Mapping 8 (SPM8: http://www.fil.ion.ucl.ac.uk/spm) and the Data Processing Assistant for Resting-State fMRI (http://www.rfmri.org/) toolbox in MATLAB. Data preprocessing included realignment and head motion correction (cumulative translation > 3 mm or rotation > 3°, and mean point-to-point translation > 0.15 mm or rotation > 0.1°, were excluded), spatial normalization (functional images were spatially normalized to Montreal Neurological Institute [MNI] template and resampled to 3*3*3 mm^3^) and smoothing (full-width at half-maximum = 6 mm*6 mm*6 mm).

ICA of the rs-fMRI data was performed using the Group ICA of the fMRI Toolbox V3.0 (GIFT V 3.0: http://matlab.mm.org/software/gift). Through GIFT, the CEN, SN, and DMN were identified using the Infomax algorithm. The number of independent components after dimension estimation, performed using the minimum description length criterion [51] of the three databases (rMDD-baseline, rMDD-six-month follow-up and HC), were 34, 35 and 33, respectively. We used principal component analysis to reduce the temporal dimension of the aggregate dataset, and the FastICA algorithm was used for independent components (with time courses and spatial maps) estimation. Components to be retained for further analysis were selected through the best-fit algorithm based on the standard template space [52].

All analyses of fMRI images were made using the SPM8 toolbox. Spatial networks of the CEN, SN, and DMN were compared between groups and over time. Independent-samples *t*-tests between the HC and rMDD groups, and paired-sample *t-*test within the rMDD group at baseline and follow-up were performed. One sample *t*-tests were also performed to moderate the spatial networks within each group. One sample *t*-tests results were corrected with the false discovery rate (FDR) correction (*p* = 0.05); two sample *t*-test results were corrected with the FDR correction (*p* = 0.01).

Age, years of education, gender and clinical variables were compared between groups by *t*-test, Chi-square test and Mann–Whitney *U* test (*p*<0.05) using IBM Statistical Package for the Social Sciences version 24.0 (SPSS24).

According to the two-sample *t*-test results, region of interest signals were extracted with the Resting-State fMRI Data Analysis Toolkit (http://restfmri.net/forum/), and correlations between aberrant FC and BDI and functional scores were tested by Spearman correlations at an alpha of 95% using IBM SPSS24.Correlation analysis results were corrected with FDR (*p =* 0.05) by Benjamini - Hochberg method.

In the *t*-test and correlation analysis, the results were considered significant when the corrected *p* value was less than 0.05 or 0.01.

## Results

### Demographic and clinical characteristics

There were no significant between-group differences in age, years of education, or gender (see Table 1). In the rMDD group, there were no significant differences in psychological or social functioning between baseline and follow-up, but BDI scores did decrease significantly (see Table 2).

**Table 1:** Demographic characteristics between groups

Variable	rMDD (*n* = 17)		HCs (*n* = 22)	*t/χ2/U*	*p*
Gender (males/females)		7/10	14/8	0.09	0.76
Age		37.50 ± 11.93	36.86 ± 10.31	0.18	0.86
Education		12(0)	12(4)	48.50	0.11

Note: rMDD: remitted major depressive disorder; HCs: healthy controls; *t*: *t*-test; *χ2*: Chi-square test; *U*: Mann–Whitney *U* test

**Table 2:** Clinical characteristics between groups

Variable (rMDD_2_ − rMDD_1_)	rMDD_1_ (*n* = 17)	rMDD_2_ (*n* = 17)	*t*	*p*
BDI	7.75 ± 4.52	3.62 ± 3.91	−3.26	<0.01
psychological functioning	60.82 ± 14.12	61.75 ± 17.80	0.22	0.83
social functioning	61.28 ± 11.85	64.72 ± 11.62	1.56	0.14

Note: rMDD_1_: patients with remitted major depressive disorder at baseline; rMDD_2_: patients with remitted major depressive disorder at follow-up; BDI: Beck Depression Inventory; psychological function: GQOLI-74 psychological functioning subscale; social functioning: GQOLI-74 social functioning subscale

### Within-group analyses

**Fig. 1:**
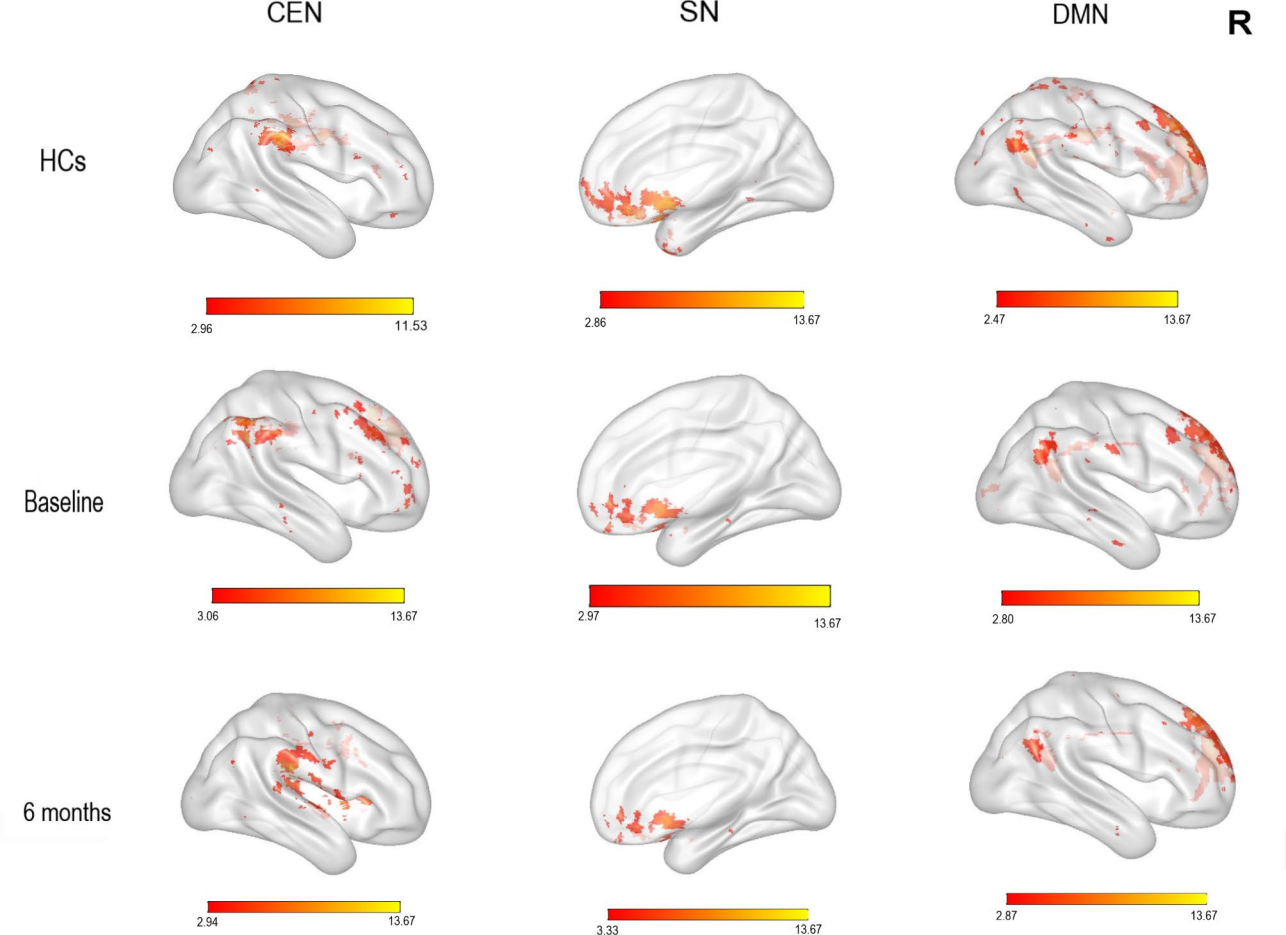
One sample *t-*test results in the CEN, SN and DMN (FDR corrected, *p* = 0.05). Colored bar represents *t*-values. CEN: central executive network; SN: salience network; DMN: default mode network; Baseline: rMDD group at baseline; 6 months: rMDD group at follow-up; HCs: healthy controls; R: right

One sample *t*-tests showed that the CEN had significantly higher FC in the DLPFC, IPL and cingulate in the HCs; in the right DLPFC and IPL in patients with rMDD at baseline; and the cingulate in patients with rMDD at follow-up (see Fig. 1, *p* = 0.05, FDR corrected). In the SN, there was higher FC in the anterior cingulate, insula, temporal pole, mPFC and supplementary motor area in both groups, and in the rMDD group at baseline and follow-up (see Fig. 1, *p* = 0.05, FDR corrected). In the DMN, there was higher FC in the mPFC, cingulate, and IPL in both groups, and in the rMDD group at baseline and follow-up (see Fig. 1, *p* = 0.05, FDR corrected).

### Between-group analyses

**Fig. 2:**
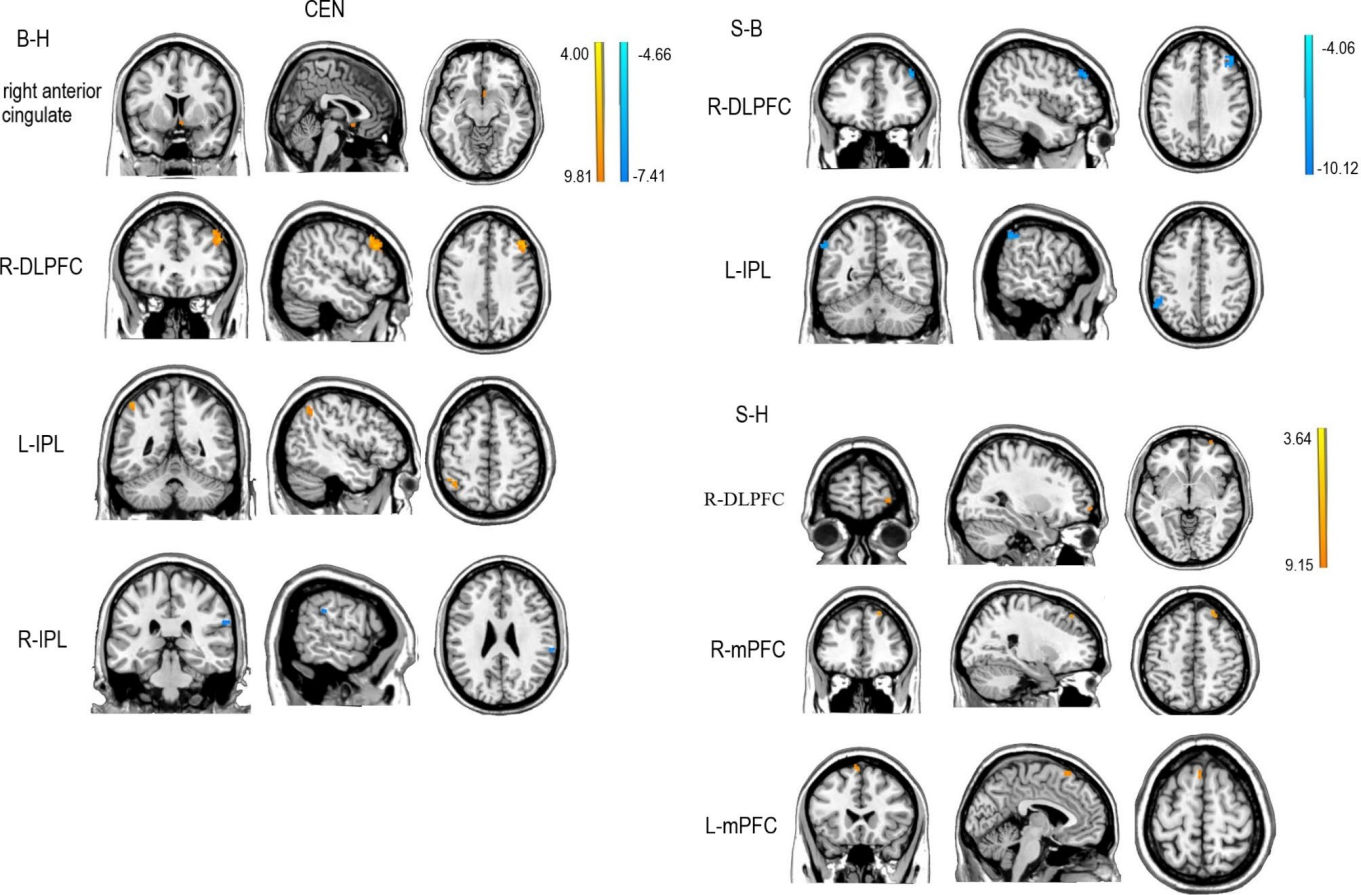
Two sample *t*-test results in the CEN (*p* = 0.01, FDR corrected). The colored bar represents *t*-values. CEN: central executive network; B-H: Two sample *t*-test results between rMDD group at baseline and HCs; S-B: Two sample *t*-test results between rMDD group at follow-up and baseline; L-IPL: left inferior parietal lobule; L-mPFC: left medial prefrontal cortex; R-IPL: right inferior parietal lobule; R-DLPFC: right dorsolateral prefrontal cortex; R-mPFC: right medial prefrontal cortex

Two sample *t*-tests showed that in the CEN, FC was higher in the right anterior cingulate, right DLFPC and left IPL, and lower in the right IPL, in patients with rMDD at baseline compared with HCs. At follow-up, FC was higher in the right DLPFC and mPFC in patients with rMDD. Compared with baseline, at follow-up, patients with rMDD had lower FC of the right DLPFC and left IPL (see Table 3; Fig. 2, *p* = 0.01, FDR corrected).

**Table 3:** Between-group comparisons of intra-network connectivity (*p* = 0.01, FDR corrected)

Group	Brain region	BA	Cluster size	*t*	Peak MNI coordinates X, Y, Z
CEN					
rMDD_1_-HC					
	right anterior cingulate	25	54	4.15	2, 8, − 10
	R-DLPFC	319	381	7.60	49, 31, 37
	L-IPL	40	348	5,52	−46, − 48, 53
	R-IPL	40	54	−5.05	62, − 31, 26
rMDD_2_ − rMDD_1_					
	R-DLPFC	9	532	−6.81	44, 38, 35
	L-IPL	40	388	−6.25	−58, − 55, 41
rMDD_2_ − HC					
	R-DLPFC	10	27	4.11	29, 65, −4
	R-mPFC	8	81	5.02	23, 38, 50
	L-mPFC	6	270	4.69	−4, 23, 62
SN					
rMDD_1_ − HC					
	L-insula	13	108	6.84	−31, 11, − 16
	left anterior cingulate	24	147	5.84	−4, 24, 27
	R-insula	47	135	5,49	32, 23, 2
	L-IPL	40	324	5,10	−61, − 40, 38
	R-precuneus	7	27	−4.26	20, − 76,44
rMDD_2_ − rMDD_1_					
	R-DLPFC	10	81	6.95	38, 59, −7
DMN					
rMDD_1_ − HC					
	R-mPFC	8	54	9.05	11, 38, 56

Note: BA: Brodmann’s area; MNI: Montreal Neuroscience Institute template; rMDD_1_: patients with remitted major depressive disorder at baseline; rMDD_2_: patients with remitted major depressive disorder at follow-up; HCs: healthy controls; CEN: central executive network; SN: salience network; DMN: default mode network; L-IPL: left inferior parietal lobule; L-insula: left insula; L-mPFC: left medial prefrontal cortex; R-IPL: right inferior parietal lobule; R-DLPFC: right dorsolateral prefrontal cortex; R-insula: right insula; R-mPFC: right medial prefrontal cortex; R-precuneus: right precuneus

**Fig. 3:**
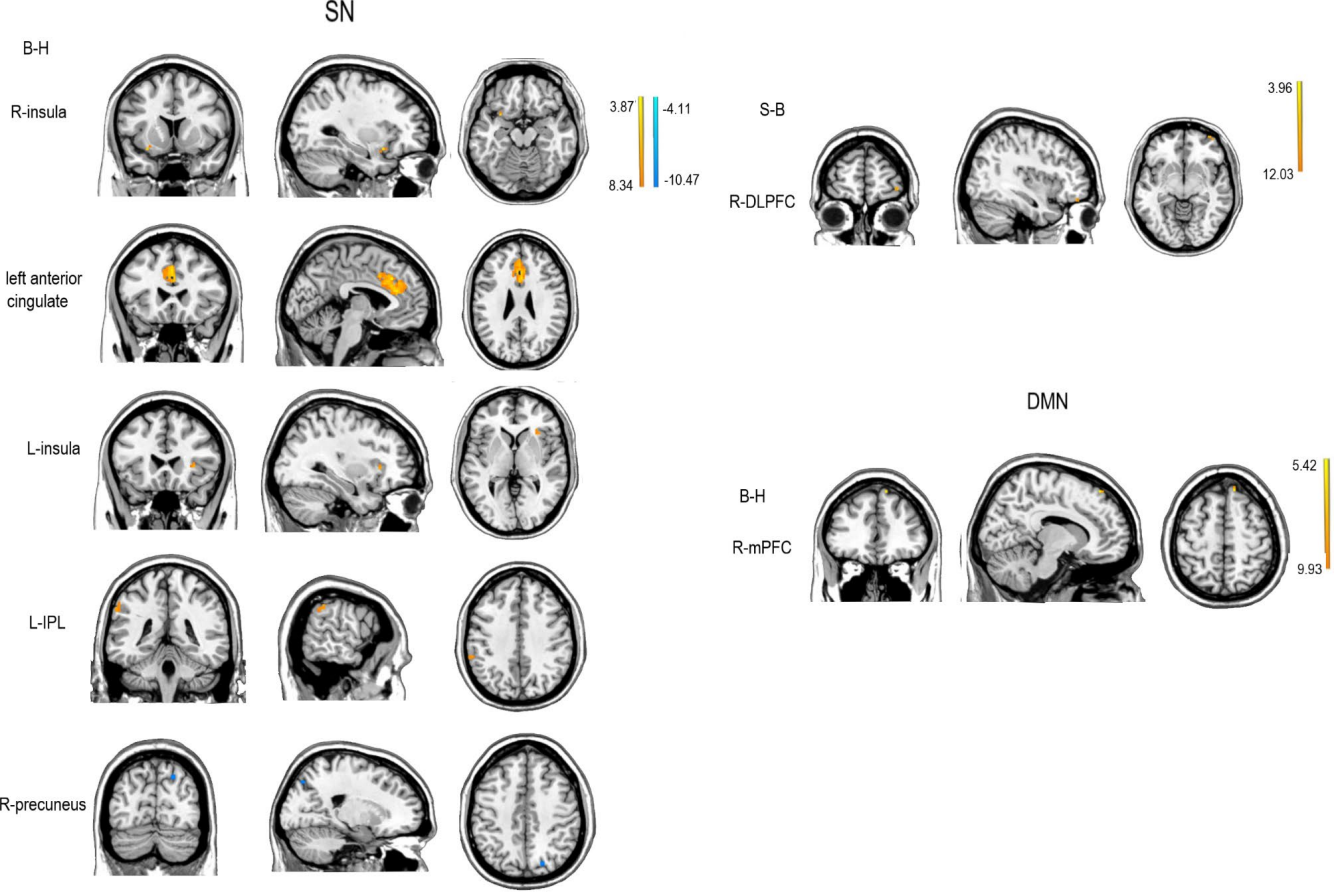
Two sample *t*-test results for the SN and DMN (*p* = 0.01, FDR corrected). The colored bar represents *t*-values. SN: salience network; DMN: default mode network; B-H: Two sample *t*-test results between rMDD group at baseline and HCs; S-B: Two sample *t*-test results between rMDD group at follow-up and baseline; S-H: Two sample *t*-test results between rMDD group at follow-up and HCs; L-IPL: left inferior parietal lobule; L-insula: left insula; R-DLPFC: right dorsolateral prefrontal cortex; R-insula: right insula; R-mPFC: right medial prefrontal cortex; R-precuneus: right precuneus

In the SN, FC was higher for the left insula, left anterior cingulate, and left IPL, and lower for the right precuneus, in patients with rMDD at baseline compared with HCs. At follow-up, there were no significant between-group differences. Patients with rMDD had higher FC of the right DLPFC at follow-up compared with baseline (see Table 3; Fig. 3, *p* = 0.01, FDR corrected).

For the DMN, FC was higher for the right mPFC in patients with rMDD at baseline compared with HCs. At follow-up, there were no significant between-group differences or differences compared with baseline (see Table 3; Fig. 3, *p* = 0.01, FDR corrected).

### Correlations analyses

**Fig. 4:**
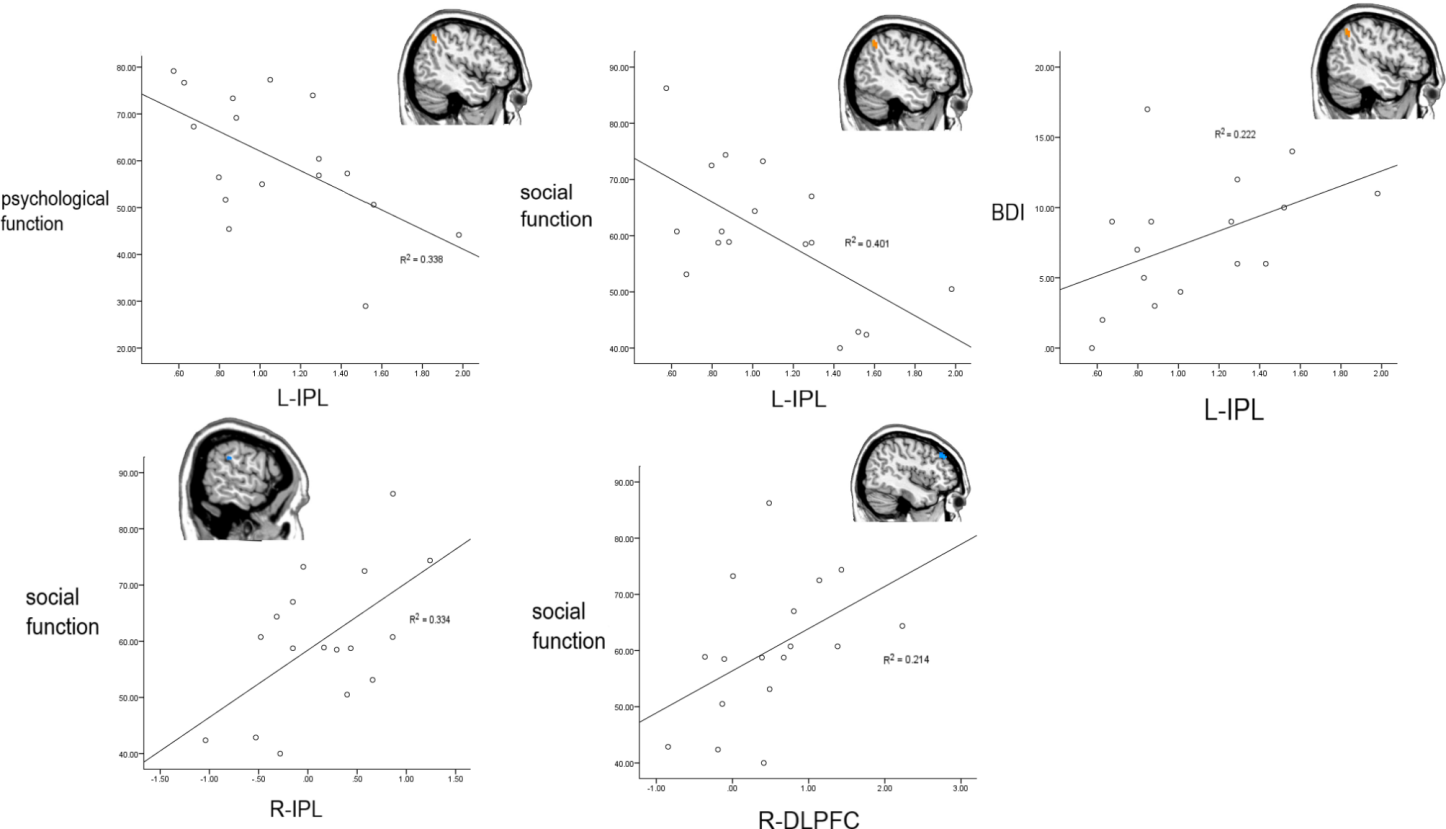
Correlations between FC and BDI, psychosocial functioning among patients with rMDD. Spearman correlations at 95% confidence level (*p* = 0.05, FDR corrected); L-IPL: left inferior parietal lobule; R-IPL: right inferior parietal lobule; R-DLPFC: right dorsolateral prefrontal cortex; BDI: Beck Depression Inventory

BDI scores were significantly, positively correlated with FC of the left IPL at baseline (see Table 4; Fig. 4, *p*<0.05, FDR corrected). FC of the left IPL was significantly, negatively correlated with the baseline social and psychological functioning subscales (see Table 4; Fig. 4, *p*<0.05, FDR corrected). FC of the right DLPFC was significantly, positively correlated with the follow-up social functioning subscale (see Table 4; Fig. 4, *p* <0.05, FDR corrected).

**Table 4:** Correlations between FC and BDI, psychosocial functioning among patients with rMDD

Brain region	BA	Cluster size	Peak MNI coordinates X, Y, Z	clinical variables	*r*	*p*
L-IPL	40	348	−46, − 48,53	psychological functioning (B)	−0.51	0.04
				social functioning (B)	−0.60	0.01
				BDI (B)	0.54	0.03
R-IPL	40	54	62, − 31,26	social functioning (B)	0.49	0.05
R-DLPFC	9	532	44,38,35	social functioning (S)	0.60	0.01

Note: Spearman correlations at 95% confidence level; (*p*=0.05, FDR corrected); MNI: Montreal Neuroscience Institute template; BA: Brodmann’s area; B: baseline; S: follow-up; L-IPL: left inferior parietal lobule; R-IPL: right inferior parietal lobule; R-DLPFC: right dorsolateral prefrontal cortex; BDI: Beck Depression Inventory

## Discussion

This study explored FC changes in the CEN, SN and DMN among patients with rMDD, and compared FC between these patients and HCs at baseline and six months later. Compared with HCs, patients with rMDD had abnormal FC within the CEN, SN, and DMN at baseline. This residual aberrant network connectivity confirms findings from previous studies [31–33].

There were anomalies in many CEN areas at the six-month follow-up; specifically, the IPL and DLPFC. Higher FC of the left IPL and right DLPFC at baseline were reduced at follow-up, and higher FC of the right IPL disappeared, possibly representing recovery from MDD. Previous studies have shown that the IPL and DLPFC are involved with self-concept and execution [53], emotional processing [54, 55], attention and working memory [56], all of which are part of psychological function. Herein, these regions also showed a close relation with social functioning. FC of the left IPL was positively correlated with BDI scores and inversely correlated with psychological and social functioning. FC of the right DLPFC was also positively correlated with social functioning. These results indicate that neural network abnormality may be one reason for social dysfunction in rMDD [45]. These correlation analyses may also represent a possible CEN-based mechanism, in which abnormal FC is associated with psychosocial functioning during early remission. FC changes may be closely related to dysfunction during recovery, so that when a degree of recovery is reached the relation between FC and psychosocial functioning ceases to be significant.

The difference between the left and right IPL in the CEN may be due to the latter’s unilaterality. The CEN consists of two symmetrical networks, with the left and right differing in function [57, 58]. Based on the results herein, the left IPL may be more closely related to Psychosocial function, though its specific relation will require further evaluation. High FC of the right DLPFC in the CEN remains six months post-remission. Previous studies have shown that the DLPFC aids ignoring distracting emotional information, being free from negative stimulation and inhibiting behaviors related to threat signals [59]. When individuals suppress unwanted thoughts and cognitive evaluations, the DLPFC is significantly activated [60]. These cumulative finding may represent a compensatory mechanism in the cognitive network of patients with rMDD, indicating that they may need more cognitive resources to fight depressive symptoms [31, 32].

In the SN and the DMN, the insula, left anterior cingulate, right precuneus, IPL, and right mPFC had abnormal baseline FC in the rMDD group. Previous studies have shown higher FC of the precuneus [61] and lower FC of the mPFC and anterior insula [25, 62]; pre-MDD remission. Since this abnormal FC of patients with MDD may fade in the opposite direction with remission, these abnormal baseline FC findings may represent rMDD. The mPFC is involved in self-referential thinking and psychological observations about others [23, 63]. The precuneus plays an important role in integrating psychological processes via cognitive control (e.g., visual representation, episodic memory, self-referential processing) [64, 65]. The insula also plays an important role in early assessment of feelings and emotions [46, 66]. The inconsistent differences among these three areas may indicate that, compared with healthy people, those with MDD may need more cognitive changes to combat their depressive symptoms. The mPFC in the DMN and the CEN, the right DLPFC in the SN and the CEN, and the left IPL and anterior cingulate in the SN and CEN all showed abnormalities and changes in FC herein, possibly indicating functional relevance among these networks [67]. Higher FC of the anterior cingulate in patients with rMDD may be a normal response during remission, since decreased connection associated with abnormal processing of mood and cognition [68] and increased FC are good predictors of remitters [59].

FC results in the CEN, SN, and DMN may mean that although there are residual symptoms at baseline, recovery is still possible. Changes in these three networks all seem to reflect improvement trends. Differences between baseline and follow-up BDI scores also suggest substantial improvement in depressive symptoms among patients with rMDD over time. This study showed that among these networks, the DMN may undergo the greatest recovery during initial remission, and that abnormalities gradually remit across remission, with only CEN abnormalities remaining after six months. This would suggest that goal-oriented cognitive control activities may play an important role in remission.

Correlation analyses herein also suggest the importance of social functioning, which current evaluation systems overlook. Although these results show that FC of the CEN is related to psychosocial functioning, symptom scores may better reflect post-remission differences compared with psychosocial functioning assessments. While it may not be appropriate to replace all symptom assessments with psychosocial functioning assessments for determining remission, this construct should certainly be considered a factor.

Further research will be needed to establish the mechanisms of rMDD recovery. To achieve this goal, it will be necessary to analyze more factors, assess patients longitudinally, and develop multifactorial evaluations. For both diagnosis and treatment, development of a more comprehensive measurement tool might be valuable, such as combining brain-level and functional symptom-level analyses, and following up on evaluation results. A more appropriate diagnostic criteria may include diagnoses and evaluations at different post-remission intervals, and organizing more comprehensive inquiry structures for those in remission. Personalized treatments should also be offered to patients at different stages.

This study was not without limitations. The sample size affects its generalizability, as does use of a six-month follow-up. The latter may have led to selection bias, by including only patients who were highly cooperative. The lack of follow-up with HC participants disallowed complete exclusion of time factors as potential influences. The lack of examining relationship between functional connectivity and residual cognitive dysfunction. The effect of drugs on the results was not taken into account. Finally, ICA confers specific limitations like subjective bias in component identification.

## Conclusions

The results herein suggest that patients with rMDD continue to have aberrant network connectivity, which changes during the initial six remission months. After this period, anomalies in the CEN remain, while those in the SN and DMN remit. Further, rs-FC of the CEN during early rMDD is associated with psychosocial functioning.

## Data Availability

The datasets used and/or analysed during the current study are available from the corresponding author on reasonable request.

## Abbreviations

MDD: major depressive disorder
rMDD: remitted major depressive disorder
HCs: healthy controls
rs-fMRI: resting-state functional magnetic resonance imaging
rs-FC: resting-state functional connectivity
DSM: Diagnostic and Statistical Manual of Mental Disorders
TR: repetition time
TE: echo time
MNI: Montreal Neurological Institute
SPSS24: Statistical Package for the Social Sciences24
CEN: central executive network
SN: salience network
DMN: default mode network
DLPFC: dorsolateral prefrontal cortex
IPL: inferior parietal lobule
mPFC: medial prefrontal cortex
ICA: independent component analysis
HAMD: Hamilton Rating Scale for Depression
GQOLI: Generic Quality of Life Inventory
BDI: Beck Depression Inventory
SPM8: Statistical Parametric Mapping 8
GIFT: Group ICA of fMRI Toolbox
FDR: false discovery rate
FC: functional connectivity
